# Effects of trigger-day progesterone in the preimplantation genetic testing cycle on the embryo quality and pregnancy outcomes of the subsequent first frozen-thawed blastocyst transfer

**DOI:** 10.3389/fendo.2023.990971

**Published:** 2023-03-06

**Authors:** Jingdi Li, Yueyue Cui, Hao Shi, Zhiqin Bu, Fang Wang, Bo Sun, Yile Zhang

**Affiliations:** ^1^Reproductive Medical Center, the First Affiliated Hospital of Zhengzhou University, Zhengzhou, China; ^2^Henan Key Laboratory of Reproduction and Genetics, First Affiliated Hospital of Zhengzhou University, Zhengzhou, China

**Keywords:** preimplantation genetic testing, trigger-day progesterone, euploid blastocyst transfer, embryo quality, pregnancy outcome

## Abstract

**Objective:**

To assess whether progesterone (P) levels on the trigger day during preimplantation genetic testing (PGT) cycles are associated with embryo quality and pregnancy outcomes in the subsequent first frozen-thawed blastocyst transfer (FET) cycle.

**Methods:**

In this retrospective analysis, 504 eligible patients who underwent ICSI followed by frozen-thawed embryo transfer (FET) with preimplantation genetic test (PGT) between December 2014 and December 2019 were recruited. All patients adopted the same protocol, namely, the midluteal, short-acting, gonadotropin-releasing hormone agonist long protocol. The cutoff P values were 0.5 and 1.5 ng/ml when serum P was measured on the day of human chorionic gonadotropin (HCG) administration, and cycles were grouped according to P level on the day of HCG administration. Furthermore, the effect of trigger-day progesterone on embryo quality and the subsequent clinical outcome of FET in this PGT population was evaluated.

**Results:**

In total, 504 PGT cycles were analyzed. There was no significant difference in the number of euploid blastocysts, top-quality blastocysts, euploidy rate, or miscarriage rate among the three groups (*P*>0.05). The 2PN fertilization rate (80.32% vs. 80.17% vs. 79.07%) and the top-quality blastocyst rate (8.71% vs. 8.24% vs. 7.94%) showed a downward trend with increasing P, and the between-group comparisons showed no significant differences (*P*>0.05). The clinical pregnancy rate (41.25% vs. 64.79%; *P*<0.05) and live birth rate (35.00% vs. 54.93%; *P*<0.05) in subsequent FET cycles were substantially lower in the high-P group than in the P ≤ 0.5 ng/ml group. After adjustments were made for confounding variables, multivariate logistic regression analysis revealed that the high-P group had a lower clinical pregnancy rate (adjusted OR, 0.317; 95% CI, 0.145–0.692; *P*=0.004) and live birth rate (adjusted OR, 0.352; 95% CI, 0.160–0.773; *P*=0.009) than the low-P group in subsequent FET cycles, and the differences were significant.

**Conclusion(s):**

This study demonstrates that in the PGT population, elevated P on the trigger day may diminish the top-quality blastocyst rate (although there is no difference in the euploidy rate). Trigger-day P is an important factor influencing clinical outcomes in subsequent FET cycles.

## Introduction

With the recent improvements in reproductive medicine theory and laboratory technology, increasing emphasis has been placed on the patient safety assessment during assisted reproductive technology (ART). High levels of progesterone (P) are not unusual in the late follicular period, and the use of gonadotropin-releasing hormone (GnRH) agonists and antagonists is known to lower these P levels. Even so, the incidences of high P among *in vitro* fertilization/intracytoplasmic sperm injection (IVF/ICSI) cycles with GnRH agonists and antagonists are reported to range from 13% to 46% (1, 2) and from 9% to 38% (3, 4), respectively. At present, the effect of high P during the late follicular phase on reproductive outcomes is controversial.

In their meta-analysis (5), Venetis et al. concluded that the current evidence does not favor an association between trigger-day P and clinical pregnancy rates. However, recent information highlights the detrimental effect of increased P on the reproductive outcomes of fresh cycles, probably because the supraphysiological levels of P affect endometrial receptivity during controlled ovarian stimulation (1, 6). Based on these findings and the development of vitrification freezing techniques, some experts have recommended a “freeze-all” strategy for these patients. In other words, embryos from fresh cycles with elevated P are frozen and transferred later in a FET cycle. This avoids the effect of controlled ovarian stimulation on the endometrium to a certain extent, but there is no consensus on its effect on embryo quality. Together with endometrial receptivity, embryo quality is a critical factor in embryo implantation (7). Several studies have agreed that P does not affect embryo quality (8–10), and this has been confirmed by data from oocyte donation (6, 11) and FET cycles (8–10). Conversely, some scholars have proposed that top-quality blastocysts represented by morphological grade are affected by high P (12, 13). Furthermore, two large retrospective studies have shown that patients with high P have lower rates of top-quality blastocysts, cumulative live birth, and implantation (14, 15). Clearly, patients with high P on the trigger day may lack top-quality blastocysts.

The rapid development of ART and genetic diagnosis technology has led to PGT. At present, PGT is classified mainly as PGT for aneuploidy (PGT-A), PGT for chromosomal structure rearrangement (PGT-SR), and PGT for monogenetic disorders (PGT-M). Patients with advanced age, recurrent pregnancy loss (RPL), and recurrent implantation failure (RIF) are the key target populations for PGT-A (16); PGT-SR is primarily utilized in the detection of chromosomal diseases, including abnormalities in the number of chromosomes, such as Klinefelter syndrome (47, XXY), and aberrant chromosomal structures such as deletion, duplication, inversion, and translocation (17); PGT-M is generally used to distinguish couples with a high risk of monogenic genetic diseases, such as common fibrocystic diseases, hereditary hemoglobinopathy, Huntington’s disease, and other rare diseases (18). PGT-A provides a relatively accurate assessment of embryo quality (19). Several clinical studies using PGT-A have found that elevated P during the late follicular period is irrelevant to the embryo euploidy rate and pregnancy outcomes (9, 10). However, these findings contradict previously reported studies that high P affects embryo quality, possibly due to the use of different thresholds (12, 15). Additionally, there are limited data assessing the correlation between P on the day of HCG injection and embryo quality, euploidy rates, and pregnancy outcomes in subsequent FET cycles. Therefore, this study aimed to measure serum P levels on the trigger day of fresh cycles and assess the impact of trigger-day P on the top-quality blastocyst rate, the euploidy rate, and clinical outcomes of the subsequent FET in the PGT population, with the aim of providing a reference for clinical work in ART.

## Materials and methods

### Study design and population

Patients who underwent preimplantation genetic test (PGT) at our center were mostly those with chromosomal translocation and monogenic diseases. This retrospective cohort study was conducted among patients who underwent ICSI/PGT for conception at the First Affiliated Hospital of Zhengzhou University Reproductive Medicine Center from December 2014 to December 2019. We included patients aged 20-40 years who accepted controlled ovarian stimulation with the unified protocol and endometrium with the same hormone replacement. Afterward, they underwent the first cycle of frozen-thawed euploid blastocyst transplantation. Owing to the retrospective nature of the study, informed consent was waived. All operations were carried out in conformity with the applicable rules and regulations.

In short, this retrospective study included 583 patients who received ICSI/PGT treatment from December 2014 to December 2019. The patient was excluded if she met any of the following criteria: recurrent spontaneous abortion; intrauterine adhesion; endometriosis; or hydrosalpinx. In the end, 504 eligible patients participated in the trial.

As described in a prior study (20), patients adopted the same protocol, namely, the midluteal, short-acting, gonadotropin-releasing hormone agonist long protocol. The dose of gonadotropin was individually coordinated according to the basic characteristics and responses of each patient. Continuous transvaginal ultrasound scans and serum estradiol (E2) and P were used to track the cycles. Triggering was employed with 250 µg recombinant human chorionic gonadotropin (r-hCG, Merck Serono, Geneva, Switzerland) and 2,000 IU u-HCG (Livzon, Guangzhou, China). Thirty-seven hours later, oocytes were retrieved under the guidance of transvaginal ultrasound.

### Laboratory procedures

All oocytes were fertilized by ICSI after 4-6 h. Morphological evaluation of blastocyst-stage embryos was conducted by experienced embryologists under equivalent laboratory conditions according to Gardner and Schoolcraft’s criteria (21). They performed whole genome amplification after trophoblast biopsy of top-quality blastocysts using a laser method. Next, aneuploidy was detected by SNP microarray chip detection technology (SNP array) or next-generation sequencing (NGS) technology for comprehensive chromosome screening (22). Euploid blastocysts with a well-expanded blastocyst cavity (B3-B5 stages), inner cell mass (grade A or B), and trophectoderm (grade A or B) were interpreted as top-quality blastocysts. We stored the blastocysts after biopsy in liquid nitrogen.

### FET endometrial preparation

The HRT protocol was adopted for the preparation of the endometrium in all thawing cycles. Subsequently, euploid blastocysts of the highest morphological grade were selected for transfer. The details and operation methods of the protocol have been published previously (23).

### P assessment immunoassay

Sex hormone concentrations were evaluated on days 2-4 of the menstrual cycle (before stimulation) and on the trigger day. We recorded the levels of anti-Müllerian hormone (AMH), estradiol (E2), follicle-stimulating hormone (FSH), and progesterone. Serum P was measured on the day of HCG administration using a validated electrochemiluminescence immunoassay (Cobas 12145383). The detection limit and sensitivity of the method were 0.03 ng/mL and 0.15 ng/mL, respectively. The intra-assay and interassay coefficients of variation were 3.0 and 5.5%, respectively. The same detection method was utilized throughout the study and calibrated regularly to reduce unnecessary errors.

### Main outcome measures

The outcomes included various indicators of embryo development and the outcomes of pregnancy. The key results of the study were the clinical pregnancy rate and live birth rate, with other indicators being the euploidy rate and the top-quality blastocyst rate. Clinical pregnancy was defined as one or more gestational sacs detected by ultrasound. Live birth was defined as the delivery of a live infant after 22 weeks of gestation. Miscarriage was defined as a spontaneous abortion of an intrauterine pregnancy before 22 weeks (24). Other indicators were as follows: oocyte maturity rate (number of MII oocytes per oocyte); 2PN fertilization rate (number of 2PN oocytes per MII oocyte); blastocyst formation rate (number of blastocysts formed per cultured); euploidy rate (number of euploid blastocysts per biopsied); and top-quality blastocyst rate (number of top-quality blastocysts per culture).

### Statistical analysis

SPSS Statistics for Windows, version 21, was used for statistical analysis. P on the trigger day was regarded as a classified variable and a continuous variable, and patients were categorized into one of the following three groups according to the P level on the trigger day: low P, defined as ≤0.50 ng/ml; medium P, defined as 0.51-1.49 ng/ml; or high P, defined as ≥1.50 ng/ml. Currently, there is no definitive cutoff value for trigger-day P, so these thresholds were chosen according to clinical practice. Studies have demonstrated that a P level above 1.50 ng/mL is the optimal threshold for observing reproductive outcomes (25, 26). However, higher P levels (> 1.5 ng/ml) have been shown to be detrimental not only to endometrial receptivity but also to embryo quality (12, 13). In addition, in a meta-analysis involving more than 55,000 cycles, the thresholds proposed by different studies for hCG-day P have ranged from 0.5 to 3.0 ng/mL, so 0.5 ng/mL was selected as the cutoff value for low P in this study (6). We also summarized each patient’s characteristics. Continuous variables are presented as the mean ± standard deviation or interquartile interval on the basis of whether they followed a normal distribution. For comparisons, Student’s t-test, one-way ANOVA, and Kruskal–Wallis tests were chosen. We use frequencies (percentages) to represent categorical variables and used chi-square tests to analyze differences between groups. We also conducted a univariate logistic analysis to explore the relationships between various variables and pregnancy outcomes. Multivariate logistic regression models were constructed for the crude and adjusted models by calculating the crude odds ratios (ORs) and adjusted ORs with 95% confidence intervals (CIs). Potential confounders were preferential and chosen by relying on ordinary clinical practice, literature, and baseline data, and adjustments were made for these confounding variables in the analysis of changes in each outcome between groups. Adjusted factors included female age at transfer, infertility duration, gravidity, parity, number of miscarriages, body mass index (BMI), basal E2, basal FSH, AMH, genetic category, endometrial thickness, days of embryonic development, E2 level on the day of HCG administration, and Gn total dose. All tests were two-sided, and statistical significance was defined as *P* < 0.05.

## Results

### Patient demographics and general characteristics

**Figure 1:**
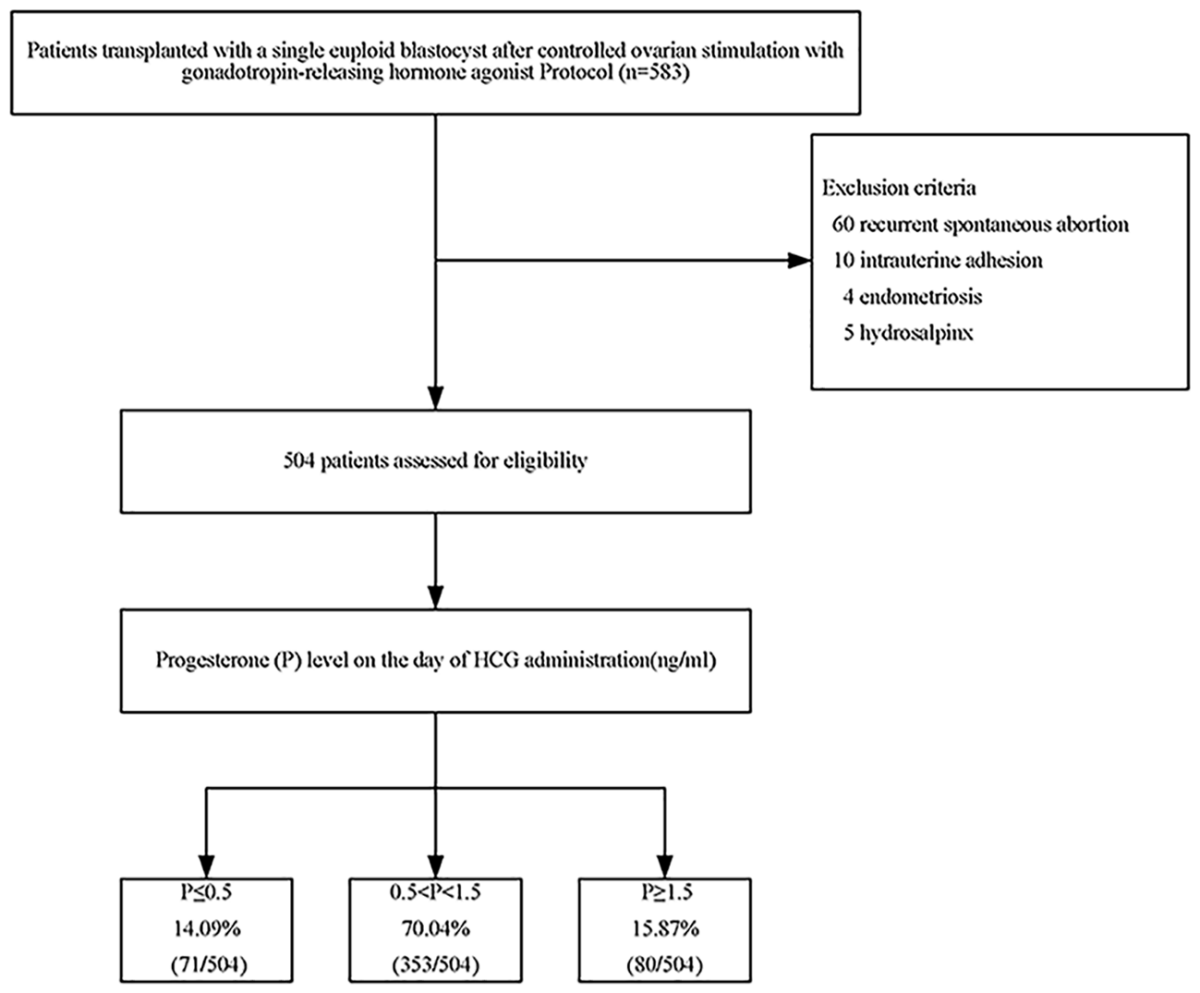
Flowchart of patients.

A total of 583 patients underwent the first FET cycles after PGT. Among them, 60 patients with recurrent miscarriages, 10 with uterine adhesions, 4 with endometriosis, and 5 with hydrosalpinx were excluded. There were 160 patients with reciprocal translocation (31.75%), 86 patients with Robertsonian translocation (17.06%), 77 patients with single-gene disease (15.28%), and 181 patients (35.91%) with different forms of chromosomal abnormalities in this study (e.g., insertion, duplication, deletion, inversion, translocation). In the end, 504 eligible patients entered the study (**Figure 1**).

Among them, 71 cases (14.09%) had serum P≤ 0.5 ng/ml, 353 cases (70.04%) had serum 0.5<P<1.5 ng/ml, and 80 cases (15.87%) had serum P≥1.5 ng/ml. There were no significant differences in the following indicators grouped by P on the trigger day: endometrial thickness, years of infertility, infertility type, gravidity, parity, number of miscarriages, genetic category, basal FSH, basal E2, AMH, days of embryonic development, and AFC. Female age increased with increasing P on the trigger day, but there were no significant between-group differences (*P*>0.05) (**Table 1**).

**Table 1:** Baseline characteristics of the population.

Characteristics	P ≤ 0.5	0.5<P<1.5	P≥1.5	*P-value* (pairwise comparisons)
N	71	353	80	–
Endometrial Thickness (mm)	11.64 ± 2.24	11.11 ± 2.52	11.35 ± 2.74	0.358
Female age at oocyte retrieval (y)	28.67 ± 4.16	29.42 ± 4.14	30.41 ± 3.69	0.120
Female age at transfer (y)	28.94 ± 4.14	29.76 ± 4.14	30.68 ± 3.77	0.120
BMI (kg/m^2^)	23.65 ± 3.58	22.91 ± 3.04	22.31 ± 2.80	0.027^†^ (0.160; 0.022; 0.401)
Infertility duration (y)	2 (1-4)	2 (1-3)	2 (1-4)	0.868
Infertility type (%)				0.888
Primary	25 (35.21%)	127 (35.98%)	31 (38.75%)	
Secondary	46 (64.79%)	226 (64.02%)	49 (61.25%)	
Gravidity	1 (0-2)	1 (0-2)	1 (0-2)	0.750
Parity	0 (0-1)	0 (0-0)	0 (0-1)	0.533
No. of miscarriages	1 (0-2)	1 (0-2)	1 (0-1)	0.372
Genetic category (%)				0.541
Reciprocal translocation	19 (26.76%)	119 (33.71%)	22 (27.50%)	
Robertsonian translocation	12 (16.90%)	55 (15.58%)	19 (23.75%)	
Single gene disorders	10 (14.09%)	55 (15.58%)	12 (15.00%)	
Others	30 (42.25%)	124 (35.13%)	27 (33.75%)	
Basal serum FSH (mIU/ml)	6.32 (5.15-7.35)	6.39 (5.29-7.23)	6.36 (5.13-7.22)	0.794
Basal serum E2 (pg/ml)	37.06 (22.72-65.72)	35.39 (24.75-47.27)	35.17 (26.96-46.14)	0.400
AMH (ng/ml)	4.29 (2.55-6.61)	4.73 (2.74-5.73)	4.13 (2.78-5.39)	0.967
Stimulation days (day)	9.99 ± 1.51	10.79 ± 1.44	11.18 ± 1.34	0.001^*†^ (<0.001; <0.001; 0.122)
Gn total dose (IU)	1897 ± 698	2068 ± 758	2314 ± 666	0.014^†^ (0.235; 0.011; 0.136)
E2 level on the day of HCG administration (pg/ml)	4551 ± 2482	6245 ± 2718	7731 ± 3377	<0.001^*†‡^ (<0.001; <0.001; <0.001)
Progesterone level on the day of HCG administration (ng/ml)	0.34 ± 0.12	0.95 ± 0.26	2.00 ± 0.41	<0.001^*†‡^ (<0.001; <0.001; <0.001)
Day of embryo development at transfer (%)				0.155
D5	46 (64.79%)	263 (74.50%)	54 (67.50%)	
D6	25 (35.21%)	90 (25.50%)	26 (32.50%)	
AFC	16.79 ± 7.12	16.69 ± 6.03	16.72 ± 4.94	0.846
No. of retrieved oocytes	15.47 ± 8.73	18.67 ± 7.24	22.27 ± 8.11	<0.001^*†‡^ (0.003; <0.001; 0.001)
No. of MII oocytes	13.26 ± 7.88	16.01 ± 6.67	18.22 ± 6.70	<0.001^*†‡^ (0.007; <0.001; 0.026)
PMOI	0.02 (0.02-0.05)	0.06 (0.05-0.09)	0.11 (0.08-0.16)	<0.001^*†‡^ (<0.001; <0.001; <0.001)
P/E2	0.07 (0.05-0.13)	0.16 (0.11-0.22)	0.27 (0.19-0.37)	<0.001^*†‡^ (<0.001; <0.001; <0.001)

BMI, body mass index; Others: Other chromosomal genetic disorders include chromosomal deletions, inversions, insertions, chimerism; FSH, follicle-stimulating hormone; E2, estradiol; AMH, anti-Müllerian hormone; Gn, gonadotropin; HCG, human chorionic gonadotropin; AFC, antral follicle count; MII, metaphase II; PMOI, progesterone to mature oocytes index; P/E2, progesterone-to-estradiol ratio.

P < 0.05 for the following pairwise comparisons: * ≤0.50 vs 0.51–1.49, †≤0.50 vs ≥1.50 or ‡ 0.51–1.49 vs ≥1.50.

### Evaluations of differences between groups

The results indicated that the total Gn stimulation days and cumulative dose were significantly higher in the high-P group at different P levels. The between-group comparisons among the three groups revealed that with increasing P on the trigger day, there was a significant upward trend in peak E2 levels, the number of oocytes retrieved, MII oocytes, PMOI, and P/E2, and there were significant differences in all between-group comparisons (**Table 1**).

As implied in **Table 2**, the three groups had statistically comparable numbers of euploid blastocysts, top-quality blastocysts, euploidy rates, and miscarriage rates (*P*>0.05). The between-group comparisons showed that the oocyte maturity rate and blastocyst formation rate decreased gradually with increasing P on the trigger day. Specifically, the oocyte maturity rate was significantly higher in the low-P and medium-P groups than in the high-P group (85.78% vs. 81.90%, *P*=0.007, 85.36% vs. 81.90%, *P*<0.001), and the blastocyst formation rates in the medium- and high-P groups were significantly lower than that in the low-P group (47.95% vs. 57.72%, *P*<0.001, 46.76% vs. 57.72%, *P*<0.001). Additionally, we noticed that the 2PN fertilization rate (80.32% vs. 80.17% vs. 79.07%) and top-quality blastocyst rate (8.71% vs. 8.24% vs. 7.94%) decreased with increasing P, although there were no significant differences between groups (*P*>0.05). Regarding the pregnancy rates of the different subgroups, the clinical pregnancy rate was significantly higher in the low-P group than in the medium-P (64.79% vs. 49.01%, *P*=0.019) and high-P groups (64.79% vs. 41.25%, *P*=0.005), albeit the difference between the medium- and high-P groups was not significant (*P*>0.05). The difference in live birth rate between the low- and high-P groups was also significant (54.93% vs. 35.00%, *P*=0.021), but that between the low- and medium-P groups or between the medium- and high-P groups was non-significant (*P* > 0.05). Interestingly, the clinical pregnancy rate (64.79% vs. 49.01% vs. 41.25%) and live birth rate (54.93% vs. 43.91% vs. 35.00%) appeared to drop linearly with increasing P.

**Table 2:** Cycle outcomes according to the Progesterone level on the day of HCG administration.

Variable	P ≤ 0.5	0.5<P<1.5	P≥1.5	*P-value* (pairwise comparisons)
No. of euploid blastocysts	1 (1-3)	2 (1-3)	2 (1-4)	0.629
No. of top-quality blastocysts	1 (0-1)	1 (0-1)	1 (0-1)	0.898
Oocyte maturation rate (%)	935 (85.78%)	5627 (85.36%)	1457 (81.90%)	0.001^†‡^ (0.746; 0.007; <0.001)
2PN Fertilization rate (%)	751 (80.32%)	4511 (80.17%)	1152 (79.07%)	0.623
Blastocyst formation rate (%)	411 (57.72%)	1973 (47.95%)	477 (46.76%)	<0.001^*†^ (<0.001; <0.001; 0.506)
Euploidy rate (%)	181 (44.04%)	861 (43.64%)	224 (46.96%)	0.421
Top-quality blastocysts rate (%)	62 (8.71%)	339 (8.24%)	81 (7.94%)	0.848
Clinical pregnancy rate (%)	46 (64.79%)	173 (49.01%)	33 (41.25%)	0.012^*†^ (0.019; 0.005; 0.218)
Live birth rate (%)	39 (54.93%)	155 (43.91%)	28 (35.00%)	0.048^†^ (0.092; 0.021; 0.168)
Miscarriage rate (%)	7 (15.22%)	18 (10.40%)	5 (15.15%)	0.269

P < 0.05 for the following pairwise comparisons: ^*^ ≤0.50 vs 0.51–1.49, ^†^≤0.50 vs ≥1.50 or ^‡^ 0.51–1.49 vs ≥1.50.

### Association between progesterone levels and pregnancy outcomes

The initial, univariate regression analysis showed that age, gravidity, number of miscarriages, P on the trigger day, days of embryonic development, and number of euploid blastocysts were the associated factors affecting pregnancy outcomes (*P*<0.05) (**Table 3**). Subsequently, we utilized a logistic regression model to evaluate the relationship between P on the trigger day and pregnancy outcomes. In an unadjusted analysis of the pregnancy outcomes in our cohort, patients were subdivided into three groups according to P at the time of triggering. With the low-P group as the reference, P was found to be significantly associated with the clinical pregnancy rate and live birth rate (*P*<0.05). To avoid potential deviation in the results and to further estimate the effects of confounding factors and P on pregnancy outcomes, we adjusted for female age at transfer, infertility duration, gravidity, parity, number of miscarriages, BMI, basal E2, basal FSH, AMH, genetic category, endometrial thickness, days of embryonic development, E2 on the trigger day, and Gn total dose. In the fully adjusted model, with trigger-day P used as a categorical variable, a considerably decreased clinical pregnancy rate for subsequent FET cycles was observed in the medium- and high-P groups compared with the low-P group (adjusted OR, 0.457; 95% CI, 0.255–0.818; *P*=0.008; adjusted OR, 0.317; 95% CI, 0.145–0.692; *P*=0.004). Regarding the live birth rate, with the low-P group used as the reference, the high-P group showed a declining trend in live birth rate in subsequent FET cycles, and the difference was significant (adjusted OR, 0.352; 95% CI, 0.160-0.773; *P*=0.009). Additionally, the difference in the live birth rate between the medium-P and low-P groups was not significant (adjusted OR, 0.571; 95% CI, 0.322-1.010; *P*=0.054). Furthermore, when trigger-day P was a continuous variable, a significant correlation (*P*<0.05) was reported between P and the clinical pregnancy rate and live birth rate in the subsequent FET cycles regardless of whether the model was adjusted (*P*<0.05) (**Table 4**).

**Table 3:** Effects of influencing factors on pregnancy outcomes by univariate logistic analysis.

	Clinical pregnancy rate		Live birth rate	
Covariate	OR (95%CI)	*P-value*	OR (95%CI)	*P-value*
Endometrial Thickness (mm)	1.001 (0.933-1.073)	0.986	0.995 (0.927-1.067)	0.887
Female age at oocyte retrieval	0.943 (0.903-0.986)	0.009	0.940 (0.899-0.982)	0.006
Female age at transfer (y)	0.942 (0.902-0.985)	0.008	0.942 (0.901-0.985)	0.008
BMI (kg/m^2^)	1.015 (0.958-1.074)	0.619	1.011 (0.955-1.071)	0.710
Infertility duration (y)	0.967 (0.897-1.042)	0.375	0.931 (0.861-1.007)	0.073
Infertility type (%)				
Primary	Reference			
Secondary	1.090 (0.758-1.567)	0.643	1.058 (0.733-1.525)	0.764
Gravidity	1.129 (0.992-1.285)	0.066	1.188 (1.043-1.354)	0.009
Parity	1.072 (0.773-1.487)	0.677	1.108 (0.798-1.538)	0.540
No. of miscarriages	1.145 (0.979-1.339)	0.090	1.221 (1.043-1.429)	0.013
Genetic category (%)				
Reciprocal translocation	Reference		Reference	
Robertsonian translocation	0.845 (0.500-1.428)	0.530	1.143 (0.671-1.949)	0.623
Single gene disorders	1.377 (0.793-2.388)	0.256	1.540 (0.869-2.726)	0.139
Other	0.768 (0.502-1.177)	0.226	0.842 (0.550-1.288)	0.428
Basal serum FSH (mIU/ml)	1.000 (0.998-1.001)	0.526	1.000 (0.998-1.001)	0.559
Basal serum E2 (pg/ml)	1.000 (1.000-1.000)	0.591	1.000 (1.000-1.000)	0.990
AMH (ng/ml)	1.037 (0.979-1.099)	0.217	1.016 (0.959-1.076)	0.590
Stimulation days (day)	0.963 (0.855-1.683)	0.528	0.925 (0.821-1.043)	0.205
Gn total dose (IU)	1.000 (1.000-1.000)	0.349	1.000 (1.000-1.000)	0.534
E2 level on the day of HCG administration (ng/ml)	1.000 (1.000-1.000)	0.529	1.000 (1.000-1.000)	0.615
Progesterone level on the day of HCG administration (ng/ml)	0.675 (0.491-0.929)	0.016	0.651 (0.468-0.907)	0.011
Day of embryo development at transfer (%)				
D5	Reference		Reference	
D6	0.634 (0.428-0.939)	0.023	0.585 (0.391-0.875)	0.009
AFC	1.015 (0.986-1.045)	0.309	1.005 (0.977-1.035)	0.723
No. of retrieved oocytes	1.000 (0.978-1.022)	0.969	0.996 (0.975-1.019)	0.750
No. of MII oocytes	1.005 (0.980-1.030)	0.692	0.998 (0.974-1.024)	0.897
No. of euploid blastocysts	1.099 (1.016-1.189)	0.018	1.097 (1.016-1.184)	0.018
No. of top-quality blastocysts	1.059 (0.913-1.228)	0.452	1.069 (0.922-1.240)	0.376

BMI, body mass index; Others: Other chromosomal genetic disorders include chromosomal deletions, inversions, insertions, chimerism; FSH, follicle-stimulating hormone; E2, estradiol; AMH, anti-Müllerian hormone; Gn, gonadotropin; HCG, human chorionic gonadotropin; AFC, antral follicle count; MII, metaphase II; PMOI, progesterone to mature oocytes index; P/E2, progesterone-to-estradiol ratio; CI, confidence interval; OR, odds ratio.

**Table 4:** Multivariate logistic regression models regarding the pregnancy outcomes.

	Crude model		Adjusted model	
Pregnancy Outcomes	OR (95%CI)	*P-value*	OR (95%CI)	*P-value*
Clinical pregnancy rate				
Progesterone level on the day of HCG administration	0.675 (0.491-0.929)	0.016	0.603 (0.404-0.901)	0.014
Groups of progesterone levels on the day of HCG administration				
P ≤ 0.5	Reference		Reference	
0.5 <P<1.5	0.522 (0.308-0.887)	0.016	0.457 (0.255-0.818)	0.008
P≥1.5	0.382 (0.197-0.738)	0.004	0.317 (0.145-0.692)	0.004
Live birth rate				
Progesterone level on the day of HCG administration	0.651 (0.468-0.907)	0.011	0.539 (0.355-0.818)	0.004
Groups of progesterone levels on the day of HCG administration				
P ≤ 0.5	Reference		Reference	
0.5 <P<1.5	0.642 (0.382-1.072)	0.091	0.571 (0.322-1.010)	0.054
P≥1.5	0.442 (0.229-0.851)	0.015	0.352 (0.160-0.773)	0.009

CI, confidence interval; OR, odds ratio.

Crude model, No adjustments for other covariates.

Adjusted model, adjusted for female age at transfer, infertility duration, gravidity, parity, number of miscarriages, body mass index, basal estradiol, basal follicle-stimulating hormone, anti-Müllerian hormone, genetic category, endometrial thickness, days of embryonic development, estradiol level on the day of HCG administration, Gn total dose.

## Discussion

The findings of this study showed that trigger-day P was independent of the embryo euploidy rate in the PGT population, that the rate of top-quality blastocysts decreased gradually with rising trigger-day P, and that trigger-day P is an important factor influencing clinical outcomes in subsequent FET cycles.

Our data showed that in the PGT population, the progesterone level of the late follicular phase in fresh cycles was irrelevant to the embryo euploidy rate. These findings were consistent with previously reported studies in which trigger-day P had no relationship with euploidy rates (8–10). Moreover, a robust multivariate regression analysis was conducted in this single-center study to account for the impact of various confounders on pregnancy outcomes. Increasing trigger-day P was thought to be critical in lowering the clinical pregnancy rate and live birth rate. Similarly, in a study of patients who underwent FET, Pal et al. confirmed that high P on the day of HCG was related to the decreased success rate of FET (27). However, these results contradicted previous studies on late-follicular P levels and FET results (1, 6, 9). The current study obtained different results from other studies regarding the relationship of elevated P with embryo quality and pregnancy outcomes, and this may be due to differences in the P cutoff value and detection methods used. For example, Bosch et al. (25) observed by trend analysis that when the serum P level on the trigger day was 1.5 ng/ml, it reached the critical threshold level at which P would have a negative effect on pregnancy outcomes. Racca et al. (12) concluded that the trigger-day P 1.5 ng/ml group had considerably decreased embryo and blastocyst utilization. Similarly, S. Santos (28) et al. reported that P levels below 0.5 ng/ml on the day of HCG administration had a detrimental effect on live birth. Various studies have used different analytical methods to determine specific thresholds for elevated P. As a result, the final results obtained by applying different cutoff values were inconsistent. On the other hand, the population was composed mainly of patients who underwent PGT, most of whom had chromosomal translocations or single-gene disorders. The heterogeneity of this population itself also explains the difference from other results.

Racca et al. (29) discovered that on the trigger day, the mean number of top-quality blastocysts was lower in the P≥1.5 ng/ml group than in the P<1.5 ng/ml group. Two large retrospective studies (14, 15) also indicated that high P was relevant to a reduced rate of top-quality blastocysts. Furthermore, Vanni et al. (13) believed that when P levels reached or approached 1.5 ng/ml (>1.49), they could be considered an early warning sign of a decline in the top-quality blastocyst rate. The present study also found that the rate of top-quality blastocysts decreased gradually with rising P, although the differences among the three groups were not significant. This also indirectly implied that high P may have an impact on the top-quality blastocyst rate. Animal studies have shown that decreased follicular P can improve oocyte development *in vitro* (30). Furthermore, Valbuena et al. found that high E2 can be harmful to cleavage-stage embryos (31). The high P on the trigger day was frequently accompanied by high E2, according to findings from this study and earlier ones (6, 25, 32). Regrettably, it is unknown whether superphysiological levels of hormones will hinder the quality of human embryos. In a study published in 2021, Tokgoz et al. (33) revealed that embryo transfer at the blastocyst stage in the fresh cycle could improve pregnancy outcomes, even though the P level on the trigger day was higher than 0.85 ng/mL. Indeed, it is unclear whether blastocyst transfer can partially offset the negative effect on pregnancy outcome of elevated P on the trigger day. Since the mechanism by which high P on the trigger day impacts embryo quality is currently unknown, it is difficult to assume that trigger-day P has a residual influence on pregnancy outcomes in subsequent FET cycles by affecting blastocyst quality. Moreover, there are few studies evaluating whether low P on the trigger day disrupts pregnancy outcomes or embryo quality in subsequent FET cycles. Only one study reported the effect of P<0.8 ng/ml on euploid embryos and reproductive outcomes in subsequent FET cycles (34). Nonetheless, a large-scale meta-analysis of over 55,000 cycles noted that P was significantly and inversely associated with pregnancy outcomes when the P level reached 0.8 ng/ml or above in the late-follicular phase (6). Therefore, a different cutoff value was selected for our study based on S. Santos et al. and Arvis et al. In fresh-embryo transfer cycles, they noticed that low P (< 0.5 ng/ml) on the day of HCG administration significantly decreased live birth rates (28, 35). However, in our research, when P was <0.5 ng/ml, neither the embryo euploidy rates nor pregnancy outcomes of subsequent FET cycles were affected. This indicates that low P in the late follicular phase will not have an impact on embryo quality. However, it may lead to changes in endometrial receptivity and thus affect pregnancy outcomes in fresh cycles. Moreover, the underlying mechanism for the high P on the trigger day during ART is not clear. Studies have shown that three main aspects contributing to high P include the number of follicles, the gonadotropin dose and actions on granulosa cells, and the role of luteinizing hormone stimulation on follicular membrane cells (36). In our research, we also noticed higher gonadotropin doses, estradiol concentrations, and retrieved and mature oocytes in the high-P group.

Our study precisely investigated the influence of low P on the embryo euploidy rate and pregnancy outcomes in subsequent FET cycles and discovered that low P was not linked to the embryo quality or pregnancy outcomes in subsequent first FET cycles. Additionally, a unified ovarian stimulation and endometrial preparation protocol was implemented in all patients. The research involved only patients who underwent single euploid blastocyst transfer. Furthermore, a standardized protocol and the application of multiple regression logistic analysis models were used to weaken the effects of potential confounding factors and ensure the reliability of the results.

However, due to the limitations of this retrospective study, there are inevitable deviations even though the effects of potential confounders were minimized. First, PGT patients in our center comprise mainly chromosomal translocations and single-gene disorders, and the results of this study cannot be extrapolated to the general infertile population. Second, it is evident from the present study that there is a gradual increase in female age with increasing P on the trigger day, and there is no denying the influence of female age on pregnancy outcomes (37). Third, there is no definitive cutoff value for trigger-day P. P assays and their cutoff values vary among studies in different centers and may vary depending on the assay method. Finally, studies have shown that the duration of P elevation also affects pregnancy outcomes (38, 39), but we did not separately analyze patients with different durations of P elevation.

In summary, we used an accurate PGT/FET cycle model to determine the relationship of trigger-day P with embryo quality and subsequent pregnancy outcomes. The results showed that in the single vitrified frozen euploid blastocyst transfer cycle, trigger-day P was irrelevant to the embryo euploidy rate. However, elevated P may reduce the rate of top-quality blastocysts. Trigger-day P is an important factor influencing pregnancy outcomes in subsequent FET cycles. Since this study includes all PGT patients subjected to the short-acting, gonadotropin-releasing hormone agonist long protocol, it remains unclear whether findings can be extrapolated to populations using other protocols or to the general infertile population. On the other hand, in view of the small sample size included in the low-P and high-P groups in this study, the existence of bias cannot be ruled out, and the lack of subsequent multicycle follow-up makes it impossible to explain the impact of high P on prognosis. Further studies and randomized clinical trials with larger sample sizes are advisable. In conclusion, we should treat this result with caution. In daily clinical practice, each center needs to evaluate its P threshold before performing FET in patients showing high P on the trigger day to obtain better outcomes.

## Data availability statement

The original contributions presented in the study are included in the article/supplementary material. Further inquiries can be directed to the corresponding author.

## Author contributions

YZ, JL and YC contributed to the study design, data analysis and manuscript preparation. HS, ZB, FW and BS handled patient recruitment and data collection. All authors contributed to the article and approved the submitted version.
